# Safety and effectiveness of adalimumab in a clinical setting that reflects Canadian standard of care for the treatment of rheumatoid arthritis (RA): Results from the CanACT study

**DOI:** 10.1186/1471-2474-12-261

**Published:** 2011-11-17

**Authors:** Boulos Haraoui, Alfred Cividino, Jacqueline Stewart, Benoît Guérette, Edward C Keystone

**Affiliations:** 1 Department of Rheumatology, CHUM-Hôpital Notre Dame, Montreal, Quebec, Canada; 2 Department of Medicine, Division of Rheumatology, McMaster University, Hamilton, Ontario, Canada; 3 Department of Medicine, Penticton Regional Hospital, British Columbia, Canada; 4 Rheumatology, Global Medical Affairs, Abbott, Rungis, France; 5 The Rebecca MacDonald Centre for Arthritis and Autoimmune Disease, Mount Sinai Hospital, Toronto, Ontario, Canada

## Abstract

**Background:**

This multicenter, open-label, prospective, single cohort study evaluated the effectiveness and safety of adalimumab in a clinical setting reflecting the Canadian standard of care for the treatment of patients with rheumatoid arthritis (RA).

**Methods:**

Patients ≥ 18 years of age with a history of active RA ≥ 3 months and fulfilling Canadian requirements for biological therapy received adalimumab 40 mg subcutaneously every other week for 12 weeks. Pre-study DMARD treatment regimens, corticosteroids, or NSAIDs were allowed throughout the study. The primary effectiveness outcome measure was the mean change in 28-joint disease activity score (DAS28) from baseline to Week 12. Secondary measures included the proportion of patients achieving joint remission (DAS28 < 2.6) and low-disease activity (DAS28 < 3.2) at Week 12, and European League Against Rheumatism (EULAR: moderate and good) and American College of Rheumatology (ACR: ACR20, 50, and 70) responses, as well as responses in ACR core components at Weeks 4, 8, and 12. Subgroup analysis included a comparison of patients naïve to biological DMARD (BDMARD) therapy versus BDMARD-experienced patients. Safety was assessed in terms of adverse and serious adverse events.

**Results:**

A total of 879 patients (mean disease duration > 12 years) were enrolled; 772 (87.9%) completed the 12-week period. Adalimumab treatment was associated with rapid and sustained improvements in the signs and symptoms of RA. Significant improvements in mean DAS28 score were observed as early as Week 4. After 12 weeks of adalimumab treatment, 15.3% and 28.9% of patients achieved clinical remission and low-disease activity, respectively. Similarly, significant improvements in ACR core components were observed as early as Week 4, with continued improvements occurring through 12 weeks. Patients naïve to BDMARD therapy demonstrated numerically greater clinical responses when compared with patients who had experienced prior BDMARD therapy, although both subgroups were associated with significant improvements from baseline. The rates and types of adverse events, as well as the results of laboratory measures, demonstrated that adalimumab was generally safe and well-tolerated.

**Conclusions:**

This study demonstrated that, under conditions reflective of the normal clinical practice in Canada, adalimumab is an effective and safe treatment for patients with RA.

**Trial registration:**

NCT00649545.

## Background

Rheumatoid arthritis (RA) is the most common inflammatory form of arthritis, affecting approximately 1% of Canadian adults [1]. The long-term prognosis of RA is poor. After 10 years, approximately 50% of patients will have work disability. Moreover, after 20 years, up to 80% of patients will have evidence of physical disability or joint abnormalities [2]. RA is also associated with premature death. Indeed, the median life expectancy of an RA population is reduced by 3 to 18 years when compared with a non-RA population [3].

Tumor necrosis factor-alpha (TNF-α), a proinflammatory cytokine, plays a critical role in mediation of the inflammatory synovitis, cartilage matrix degradation, and bony erosions in RA [4]. TNF-α has been shown to be highly expressed in inflamed synovial tissue of patients with RA, particularly at the cartilage-pannus junction [5-7]. Adalimumab is a fully human recombinant immunoglobulin G1 (IgG1) monoclonal antibody directed against TNF-α [8,9]. Structurally and functionally analogous to naturally occurring human IgG1, adalimumab has a terminal half-life of approximately 2 weeks. Adalimumab has a high affinity for TNF-α and does not bind to other cytokines such as lymphotoxin. Adalimumab exerts its therapeutic effects by blocking the interaction of TNF-α with the p55 and p75 TNF-α cell surface receptors [8].

Adalimumab has been extensively studied for the treatment of RA. The clinical efficacy and safety parameters of recommended doses of adalimumab were determined from the results of 4 pivotal trials (DE011, ARMADA, STAR and DE019) [10-13]. Data from these studies have shown that adalimumab significantly reduced the signs and symptoms of RA. The administration of adalimumab was also associated with significant improvements in physical function and quality of life-related outcomes. The beneficial effects of adalimumab were observed whether it was administered as monotherapy or used in combination with traditional disease-modifying antirheumatic drugs (DMARDs). Moreover, data from the DE019 study [11] indicated that adalimumab reduced the radiologic progression of RA when administered to patients who partially responded to methotrexate (MTX) therapy. Interestingly, the results from the DE013 (PREMIER) study [14] confirmed that the administration of adalimumab in combination with MTX is more effective than monotherapy with either adalimumab or MTX alone in inhibiting the progression of structural joint damage in patients with early RA. Results obtained from open-label-extension studies suggest that the long-term administration of adalimumab is associated with continued efficacy and is generally safe and well-tolerated [15-17].

While randomized, controlled trials (RCTs) are fundamental in assessing efficacy and tolerability of therapies, often there are discrepancies between treatments in controlled circumstances and those of usual clinical practice. In addition, practice may vary between countries and regions. The CanACT study was designed to assess the effectiveness and tolerability of subcutaneous (sc) adalimumab 40 mg administered every other week (eow) to patients with RA in a setting that reflects Canadian routine clinical care.

## Methods

### Patients

Eligible patients were 18 years of age or older and had a history of RA, diagnosed according to the 1987 revised criteria of the American College of Rheumatology (ACR) [18] for at least 3 months. To be eligible for study entry, patients had to have active disease, which was defined by the presence of at least 5 swollen joints (of 66 joints evaluated) and 1 of the following: positive rheumatoid factor (RF), 1 or more joint erosions present on x-ray, or a disability index of the health assessment questionnaire (HAQ-DI) score of at least 1. In addition, patients had to have a history of unsatisfactory responses or intolerance to prior antirheumatic therapies, as required by local Canadian provincial guidelines for the initiation of biological DMARDs (BDMARDs). Concomitant prednisone dosages had to be < 10 mg/day. All patients were screened for latent tuberculosis and treated, when indicated, according to the Canadian Rheumatology Association (CRA) recommendations.

Patients were excluded from the study if they had failed more than 1 BDMARD, had received prior anti-CD4 therapy, had a positive HIV status, or had a history of active tuberculosis or other infections suggestive of significant or profound immunosuppression. Patients with a history of cancer within the past 10 years other than successfully treated nonmetastatic cutaneous squamous cell or basal cell carcinoma and/or localized carcinoma in situ of the cervix were excluded, as were those with a history of malignant lymphoma or leukemia, those with a history of neurologic symptoms suggestive of central nervous system demyelinating disease (eg, multiple sclerosis), and those with a history of or current acute inflammatory joint disease or an origin other than RA (eg, systemic lupus erythematosus).

### Protocol

This study was a multicenter, open-label, prospective, single cohort study of adalimumab with concomitant antirheumatic medications. The primary objective was to assess the safety of adalimumab 40 mg administered eow to patients with moderate to severe, active RA who failed prior DMARD therapy. The secondary objectives of the study were to assess the clinical effectiveness of adalimumab and its impact on quality of life, productivity, and specific direct medical and indirect resource utilization in these same patients.

Performed at 69 sites across Canada, this study was conducted in accordance with ICH Good Clinical Practice and ethical principles as defined in the Declaration of Helsinki (1989 revision) and all applicable local regulations. All patients gave their written, informed consent. The study protocol, informed consent form, and patient information were reviewed and approved for community sites by an independent Ethics Committee/Institutional Review Board (IRB Services, Aurora, Ontario) or by respective University Institutional review boards.

After screening and baseline evaluations, patients returned for assessments every 4 weeks until the Week 12 visit. Patients who completed the minimum 12-week treatment period before adalimumab became commercially available were eligible to enter a 12-week continuation phase. Results are presented for the first 12 weeks of therapy only.

All patients received adalimumab at a dose of 40 mg sc eow. All patients were instructed in self-injection techniques. DMARD treatment regimens as well as prednisone or equivalent (< 10 mg/day) and nonsteroidal anti-inflammatory drugs (NSAIDs) were allowed to continue at the prestudy dose throughout the course of the study. Other biological therapies and cyclosporine were excluded during the study. Patients previously treated with approved biologics (eg, etanercept, infliximab, anakinra) were allowed to enter the study only if the respective treatment was discontinued at least 2 months prior to baseline. Cyclosporine had to be discontinued at baseline. Intra-articular injections of corticosteroids were allowed at any time during the course of the study if deemed absolutely necessary to mimic real practice. Injected joints were excluded from further evaluation.

The primary effectiveness outcome measure in this study was the mean change in the 28-joint disease activity score (DAS28) [19] from baseline to Week 12. Secondary effectiveness assessments included the proportion of patients achieving clinical remission (defined as DAS28 < 2.6) and low-disease activity (defined as DAS28 < 3.2) at Week 12, as well as the proportion achieving European League Against Rheumatism (EULAR: moderate and good) and American College of Rheumatology (ACR: ACR20, 50, and 70) responses at Weeks 4, 8, and 12. Other secondary outcome measures were the mean changes in ACR core components [tender joint count, swollen joint count, erythrocyte sedimentation rate (ESR), physician's global assessment of disease activity, patient's global assessment of disease activity, patient's assessment of pain, and physical function as assessed by the HAQ-DI [20]]. Patients who failed to achieve at least an ACR20 response at Week 12 were analyzed for mean changes and mean percent changes in ACR core components from baseline to Week 12.

Safety was assessed on the basis of adverse events reported by patients, physical examination findings, and laboratory evaluations. All adverse events were coded according to the MedDRA dictionary of terms (version 9.0). All non-serious adverse events were collected from the time of the first dose of study drug until 5 half-lives (70 days) after discontinuation of study drug. Serious adverse events were collected from the time the patient signed the study-specific informed consent until 5 half-lives after discontinuation of study drug. All serious adverse events, including those that occurred during the 70-day post-therapy period, were followed until resolution or stabilization of the event was documented. Adverse events are reported during the first 12 weeks plus the 70-day post-therapy period.

### Statistical Analysis

All patients who received at least 1 dose of adalimumab and returned for 1 or more follow up visits were included in the effectiveness analysis. This included patients who were protocol violators. Observed data analyses were used to describe the effectiveness of adalimumab at Weeks 4, 8 and 12. There were no replacements of missing data conducted prior to the Week 12 visit. Subgroup analysis included a comparison of patients who were naïve to BDMARD therapy versus patients who had experienced prior BDMARD therapy. All patients who received at least 1 dose of adalimumab were included in the safety analysis.

The statistical significance of the change in DAS28 from baseline to Week 12 of treatment was assessed with the paired Student's *t*-test. Linear regression analysis was used to estimate and test the statistical significance of the rate of change in the DAS28 over time. The proportions of patients achieving ACR20, ACR50, and ACR70 therapeutic responses at Weeks 4, 8, and 12 were calculated using the number of patients returning for follow-up at these visits as the denominator. The statistical significance of any change in the continuous-scale secondary effectiveness measures, specifically physician and patient global assessment of disease activity, patient assessment of pain, and HAQ-DI scores, was also assessed with the paired Student's *t*-test. The rate of change in these variables over time during the 12 weeks of the study was estimated and tested for statistical significance using repeated measures linear regression analysis.

To assess the potential effect of bias due to differential attrition, the baseline characteristics of the patients who reached Week 12 were compared with those of patients who did not complete the first 12 weeks of the study.

The incidence of adverse events was reported as the proportion of patients with at least 1 event and the number of events per 100 patient-years.

## Results

### Patient Disposition

A total of 879 patients were enrolled from 69 sites across Canada. Of these, 772 (87.8%) completed the 12-week study. The primary reasons for study discontinuation of the remaining 107 patients were as follows: lost to follow-up (n = 48, 5.5%), adverse events (n = 39, 4.4%), lack of efficacy (n = 15, 1.7%), withdrawal of consent (n = 4, 0.5%), and protocol violation (n = 1, 0.1%).

### Demographic and Baseline Characteristics

Demographic and disease characteristics at baseline were typical of a patient population with long-standing and moderate to severe RA (Table 1): the mean disease duration since diagnosis was 12.5 years, while the mean tender and swollen joint counts were 14.9 and 13.2, respectively, the mean HAQ-DI score was 1.5, and the mean DAS28 was 6.1.

**Table 1:** Demographic and Clinical Characteristics at Baseline

Characteristics		Enrolled patients (N = 879)
Demographics	Age, years	54.4 ± 11.5
	Female, no (%)	692 (78.7)
	White, no (%)	820 (93.3)
	Disease duration, years	12.5 ± 9.7
	RF > 20 IU/mL, no (%)	661 (75.2)
ACR core set	Tender joint count (0-68 scale)	14.9 ± 7.1
	Swollen joint count (0-66 scale)	13.2 ± 5.2
	Patient's assessment of pain, mm (0-100 mm VAS)^a^	66.2 ± 22.3
	Patient's global assessment of disease activity, mm (0-100 mm VAS)^b^	65.1 ± 22.7
	Physician's global assessment of disease activity, mm (0-100 mm VAS)^b^	63.7 ± 17.6
	HAQ-DI score (0-3 scale)^c^	1.5 ± 0.6
	DAS28 Score	6.1 ± 1.2
Acute phase reactants	ESR (mm/hr) (normal value < 20 mm/h for men, < 30 mm/h for women)	30.3 ± 23.8
	CRP (mg/L) (normal value < 10 mg/L)	24.0 ± 30.8
Quality of life	HUI2 score	0.59 ± 0.22
	HUI3 score	0.38 ± 0.29
DMARD therapy	Receiving adalimumab + 0 DMARD, no. (%)	135 (15.4)
	Receiving adalimumab + 1 DMARD, no. (%)	371 (42.2)
	Receiving adalimumab + 2 DMARDs, no. (%)	284 (32.3)
	Receiving adalimumab + ≥ 3 DMARDs, no. (%)	89 (10.1)
	Receiving methotrexate, no (%)	549 (62.5)
	Receiving leflunomide, no. (%)	248 (28.2)
	Receiving sulfasalazine, no. (%)	104 (11.8)
	Receiving hydroxychloroquine, no. (%)	248 (28.2)
	Receiving prednisone, no. (%)	381 (43.3)
	Prednisone ≤ 5 mg, no. (%)	158 (18)
	Prednisone > 5 mg, no. (%)	223 (25.3)
	Patients that received prior BDMARD therapy no. (%)	242 (27.5)
	Patients that received etanercept no. (%)	131 (54.1)
	Patients that received infliximab no. (%)	65 (26.9)
	Patients that received other BDMARD no. (%)	46 (19)

*Indicating a statistical difference (*P *< 0.05) between those that continued after the Week 12 visit and those that did not.

^a^0 = no pain and 100 = severe pain.

^b^0 = no disease activity and 100 = extreme disease activity.

^c^0 = no difficulty and 3 = unable to perform activity.

ACR: American College of Rheumatology; CRP: C-reactive protein; DAS28: 28-joint disease activity score; DMARD: disease-modifying antirheumatic drug; ESR: erythrocyte sedimentation rate; HAQ-DI: disability index of the health assessment questionnaire; HUI: health utility index; RF: rheumatoid factor; VAS: visual analog scale.

A total of 860 (97.8%) of the patients previously received treatment with 1 or more DMARDs at the time of enrollment; these agents could be continued throughout the course of the study. Of the patients enrolled, 42.2% were treated with adalimumab in combination with a single DMARD, 32.3% were treated with adalimumab in combination with 2 DMARDs, and 10.1% were treated with adalimumab in combination with ≥ 3 DMARDs. The most frequently co-administered DMARDs were MTX (62.5%), leflunomide (28.2%), hydroxychloroquine (28.2%), and sulfasalazine (11.8%). Prednisone was used concomitantly by 381 (43.3%) patients. Of these, 223 (25.3%) received > 5 mg of prednisone/day and 158 (18.0%) received ≤ 5 mg/day. Prior BDMARD therapy use was reported by 242 (27.5%) patients, 131 (54.1%) of whom had received etanercept and 65 (26.9%) of whom had received infliximab.

No differences were detected between the baseline demographic and disease characteristics of patients who reached the Week 12 visit and those who discontinued before the Week 12 visit (data not shown), with the exception of the proportion of patients that received adalimumab without a DMARD (29.9% vs. 13.3%, *P *= 0.001).

### Effectiveness

**Figure 1:**
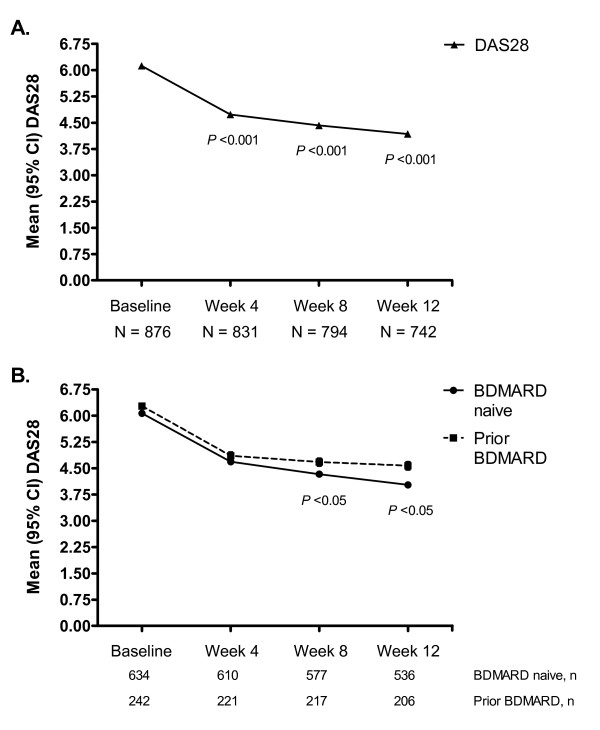
**Changes Over Time in the Mean 28-joint Disease Activity Score (DAS28)**. Mean change (95% confidence intervals [CI]) in DAS28 in patients receiving adalimumab 40 mg administered subcutaneously every other week with concomitant standard antirheumatic therapy. A.) All randomized patients. The mean DAS28 at Weeks 4, 8, and 12 were significantly lower than the mean DAS28 at baseline (*P *< 0.001). B.) Subgroup analysis of randomized patients on the basis of experience with biological DMARD (BDMARD) therapy. The mean DAS28 at Weeks 8 and 12 was significantly lower in BDMARD-naïve patients than in patients who experienced prior BDMARD therapy (*P *< 0.05).

Mean DAS28 decreased from 6.1 (95% confidence interval [CI]: 6.0, 6.2) at baseline to 4.2 (95% CI: 4.1, 4.3) at Week 12 (*P *< 0.001; Figure 1A), with a significant decrease occurring as early as Week 4. From a linear regression analysis, mean change in DAS28 (SD) was -0.157 (0.006) per week (*P *< 0.001). Following 12 weeks of treatment with adalimumab, 15.3% and 28.9% of patients achieved clinical remission (defined as a DAS28 value < 2.6) and low-disease activity (defined as a DAS28 value < 3.2), respectively.

**Figure 2:**
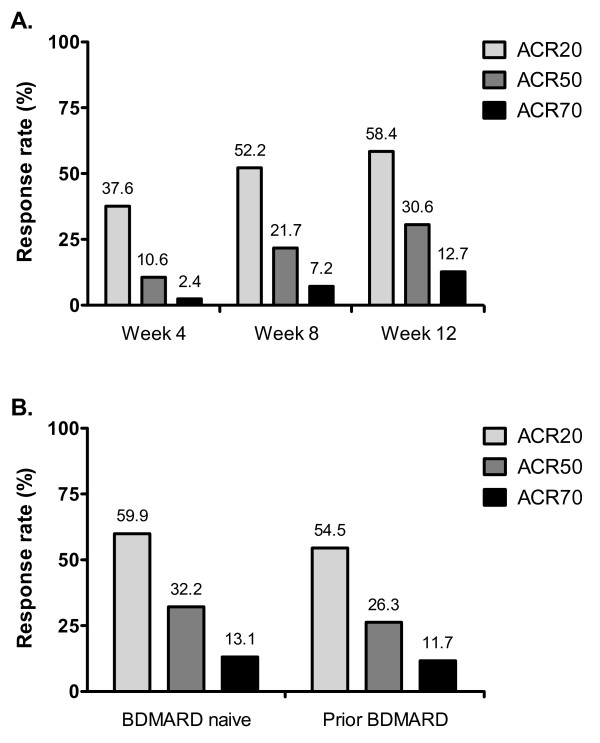
**Response in American College of Rheumatology (ACR) Criteria Following Adalimumab Treatment**. Percentages of patients who met the AC R criteria for 20%, 50%, and 70% improvement (ACR20, ACR50, and ACR70, respectively) with adalimumab 40 mg administered subcutaneously every other week with concomitant standard antirheumatic therapy. A.) All randomized patients. B) Subgroup analysis of randomized patients on the basis of experience with biological DMARD (BDMARD) therapy.

Moderate and good clinical responses, as defined by the European League Against Rheumatism (EULAR) criteria [21], were observed at Week 4 for 50.1% and 11.7% of patients, respectively (data not shown). Patients with a good EULAR response increased steadily over time, with 25.9% of patients achieving a good response at 12 weeks. Similar trends were observed for ACR response criteria. Following 4 weeks of treatment, the proportions of patients achieving ACR20, ACR50, and ACR70 improvements were 37%, 10.6%, and 2.4%, respectively (Figure 2A). These proportions increased to 58.4%, 30.6%, and 12.7% following 12 weeks of adalimumab therapy.

Patients experienced improvements in each of the ACR core components during 12 weeks of adalimumab treatment, with significant improvements occurring as early as Week 4 (Table 2). By Week 12, the mean (SD) number of tender joints decreased from 14.9 (7.1) to 6.8 (6.8), and the mean (SD) number of swollen joints was reduced from 13.2 (5.2) to 6.4 (5.2). As measured on a 0-100 mm Visual Analog Scale (VAS), patient's assessment of pain decreased from a mean (SD) of 66.2 (22.3) to 37.3 (27.5), patient's assessment of disease activity decreased from 65.1 (22.7) to 37.4 (27.2), and physician's assessment of disease activity decreased from 63.6 (17.6) to 29.0 (22.7). The mean (SD) HAQ-DI score improved by an average of 0.5 units from 1.5 (0.6) at baseline to 1.0 (0.7) at Week 12. In addition, significant reductions in ESR were observed as early as Week 4, resulting in near normal levels. At Week 12, the ESR decreased from a mean (SD) of 30.3 (23.8) mm/h at baseline to 20.0 (18.6) mm/h (*P *< 0.001). Repeated measures linear regression analysis demonstrated a significant change over time in each of the ACR core components (*P *< 0.001, data not shown). A subset analysis of patients who failed to achieve at least an ACR20 response at Week 12 (n = 321/772, 41.6%) demonstrated reductions in ACR core component values at Week 12 (Table 3). Furthermore, ACR20 non-responders demonstrated > 20% improvements from baseline in tender and swollen joint counts and in physician's global assessment of disease activity.

**Table 2:** American College of Rheumatology Core Components and Acute Phase Reactants

ACR Core Reactant	Components and Acute Phase	Baseline	Week 4	Week 8	Week 12
**ACR Core Set**	Tender joint count (0-68 scale)	14.9 ± 7.1	9.4 ± 7.1*	7.8 ± 7.1*	6.8 ± 6.8*
	Swollen joint count (0-66 scale)	13.2 ± 5.2	8.7 ± 5.6*	7.4 ± 5.5*	6.4 ± 5.2*
	Patient's assessment of pain, mm (0-100 mm VAS)^a^	66.2 ± 22.3	45.0 ± 26.4*	40.3 ± 26.9*	37.3 ± 27.5*
	Patient's global assessment of disease activity, mm (0-100 mm VAS)^b^	65.1 ± 22.7	45.1 ± 25.7*	41.0 ± 26.7*	37.4 ± 27.2*
	Physician's global assessment of disease activity, mm (0-100 mm VAS)^b^	63.6 ± 17.6	40.7 ± 22.5*	33.5 ± 22.4*	29.0 ± 22.7*
	HAQ-DI score (0-3 scale)^c^	1.5 ± 0.6	1.2 ± 0.7*	1.1 ± 0.7*	1.0 ± 0.7*
**Acute Phase Reactant**	ESR (mm/h) normal value < 20 mm/h	30.3 ± 23.8	20.6 ± 20.1*	20.7 ± 19.7*	20.0 ± 18.6*

*P < 0.001 vs. baseline.

^a^0 = no pain and 100 = severe pain.

^b^0 = no disease activity and 100 = extreme disease activity.

^c^0 = no difficulty and 3 = unable to perform activity.

ACR: American College of Rheumatology; ESR: erythrocyte sedimentation rate; HAQ-DI: disability index of the health assessment questionnaire; VAS: visual analog scale.

**Table 3:** American College of Rheumatology Core Components and Acute Phase Reactants for Patients Identified as ACR20 Non-responders at Week 12

ACR Core Reactant	Components and Acute Phase	ACR20 Non-responder at Week 12 (N = 321/772, 41.6%)			
		**Baseline**	**Week 12**	**Change**	**% Change**

ACR Core Set	Tender joint count (0-68 scale)	14.4 ± 7.8	10.6 ± 7.9	-3.8 ± 6.2	-21.6 ± 57.3%
	Swollen joint count (0-66 scale)	12.9 ± 5.2	9.1 ± 5.7	-3.8 ± 5.0	-28.1 ± 41.4%
	Patient's assessment of pain, mm (0-100 mm VAS)^a^	64.7 ± 24.3	55.3 ± 26.3	-9.1 ± 25.1	7.1 ± 275%
	Patient's global assessment of disease activity, mm (0-100 mm VAS)^b^	64.6 ± 24.1	54.9 ± 26.6	-9.5 ± 25.1	3.3 ± 191%
	Physician's global assessment of disease activity, mm (0-100 mm VAS)^b^	63.1 ± 17.8	42.7 ± 24.1	-20.5 ± 23.0	-31.1 ± 38.2%
	HAQ-DI score (0-3 scale)^c^	1.6 ± 0.6	1.4 ± 0.7	-0.2 ± 0.4	-14.7 ± 37.1%
Acute Phase Reactant	ESR (mm/h) normal value < 20 mm/h	29.6 ± 23.7	23.5 ± 21.6	-5.9 ± 15.8	-9.2 ± 76.8%

All values are mean ± standard deviation.

^a^0 = no pain and 100 = severe pain.

^b^0 = no disease activity and 100 = extreme disease activity.

^c^0 = no difficulty and 3 = unable to perform activity.

ACR: American College of Rheumatology; ESR: erythrocyte sedimentation rate; HAQ-DI: disability index of the health assessment questionnaire; VAS: visual analog scale.

When patients were classified according to prior experience with a BDMARD, a clear trend in clinical responses was observed: patients naïve to BDMARD therapy typically demonstrated greater clinical effectiveness when compared with patients who had experienced prior BDMARD therapy, despite comparable baseline disease characteristics (data not shown). For example, BDMARD-naïve patients exhibited lower DAS28 scores at Week 12 than patients who had experienced prior BDMARD therapy (4.0 versus 4.6 for BDMARD-naive and prior BDMARD subgroups, respectively; Figure 1B). Similarly, patients naive to BDMARD therapy demonstrated numerically higher rates of ACR20, 50, and 70 responses at Week 12 when compared with patients who had experienced prior BDMARD therapy (Figure 2B).

### Safety

A total of 551 non-serious adverse events were reported by 371(43.4%) patients during the safety assessment period, translating into an overall rate equivalent of 2.6 events/patient-year. The majority of the adverse events were mild to moderate in intensity. Adverse events that occurred in more than 1% of the study population are summarized in Table 4. Injection site reaction was the most common adverse event, reported by 9.9% of the patients. The most common injection site reaction was injection site erythema, which was reported by 3.5% of the patients; injection site reactions described as pruritus or rash were reported by 1.4% and 2.8% of patients, respectively. Of the treatment-emergent adverse events considered by the investigator to be related to study drug, injection site reaction and headache were the most frequently reported (≥ 5% of patients). One patient reported 2 breast cysts following 37 days of treatment with adalimumab, which, although severe, were deemed to be non-serious and unrelated to study drug.

**Table 4:** Proportion and Rates of Adverse Events that are Probably or Possibly Related to Adalimumab Observed in More Than 1% of the Patients

Adverse events observed in > 1% of the patients (probably or possibly related to adalimumab)	No. of Events	No. of Patients	Patients (%)	Events per 100 patient-years
Injection site reaction NOS^a^	145	87	9.9	39.0
Headache	63	46	5.2	16.9
Injection site erythema	55	31	3.5	14.8
Nausea	38	26	3	10.2
Rash NOS	26	25	2.8	7.0
Diarrhea NOS	27	22	2.5	7.3
Fatigue	21	16	1.8	5.6
Upper respiratory tract infection NOS	17	16	1.8	4.6
Injection site pruritus	23	13	1.5	6.2
Dizziness	12	12	1.4	3.2
Pruritus	14	12	1.4	3.8
Rheumatoid arthritis	14	12	1.4	3.8
Nasopharyngitis	13	11	1.3	3.5
Adverse drug reaction	11	10	1.1	3.0
Injection site rash	11	10	1.1	3.0
Urinary tract infection NOS	10	9	1	2.7

^a^Defined as localized injection-site bruising, burning, dermatitis, erythema, induration, inflammation, irritation, mass, edema, pain, pruritus, rash, stinging, swelling, and warmth.

NOS: not otherwise specified.

A total of 19 serious adverse events were reported by 16 (1.8%) patients. The most frequently reported events were cardiac disorders (n = 2, 0.2%) and gastrointestinal disorders (n = 2, 0.2%). Specifically, these included 1 case of congestive heart failure (CHF), 1 case of mitral valve incompetence, 1 case of myocardial infarction, 1 case of pulmonary edema, 1 case of pancreatitis, and 1 case of gastroesophageal reflux disease. One patient died within the safety assessment period. The cause of death was myocardial infarction secondary to staphylococcal sepsis, attributed to study drug.

In all, 9 (1.0%) patients reported 10 incidences of serious infections within the safety assessment period. In addition to the *Staphylococcus aureus *septicemia that occurred in the patient who died, other incidences of serious infection included 3 cases of pneumonia, 2 cases of surgical wound infections, 1 case of postoperative infection, 1 case of foot cellulitis, 1 case of possible septic arthritis, and 1 case of pyelonephritis. The rate of serious infections was estimated to be 2.4 events/100 patient-years.

One patient was diagnosed with basal cell carcinoma 97 days after starting study drug. This event was considered to be serious but of moderate severity and not related to adalimumab according to the judgment of the treating physician.

Adalimumab therapy was associated with slight decreases in white blood cell count, neutrophils, monocytes, and platelet counts and with modest increases in lymphocytes and eosinophils (data not shown). However, none of these changes were considered to be clinically relevant. Mean changes in clinical chemistry parameters also were small and not clinically relevant. No patient had a clinically relevant shift in any urinalysis value. Furthermore, changes in mean vital signs (systolic and diastolic blood pressure, heart rate, respiratory rate, and body temperature) were small and clinically unremarkable (data not shown).

## Discussion

The relationship between observational studies and RCTs is widely considered to be complementary, not alternative [22]. Open-label observational studies offer greater understanding of the effectiveness of medications in real-world clinical practice, which cannot be provided by RCTs. In addition, the inclusion of patients and treatments reflecting varying clinical practices, rather than the narrow range included in RCTs, offers a better environment to study the safety profile of drugs and to determine their tolerability profile.

CanACT is the largest observational study designed to evaluate the effectiveness and safety of adalimumab in a setting reflective of the Canadian clinical practice for the treatment of RA. In this study, patients who failed prior DMARD therapy received sc adalimumab eow; patients were allowed to continue antirheumatic treatment (eg, DMARDs, low-dose corticosteroids, NSAIDs, analgesics, etc.), with the exception of BDMARDs and cyclosporine. Patients had experienced many years of active disease prior to enrollment [mean (SD) = 12.5 (9.7) years], a result that is reflective of access to care in Canada and consistent with data from many European and American BDMARD registries [23]. The results of this 12-week study support the effectiveness and tolerability of adalimumab under conditions of routine clinical care.

As demonstrated through the examined effectiveness measures, responses to adalimumab therapy were rapid. Significant reductions from baseline were observed as early as 4 weeks from the start of therapy. At this time point, more than a third (37.6%) of the patients already were classified as ACR20 responders. Significant changes in DAS28 also were observed at this visit. Interestingly, improvements in DAS28 were numerically greater during the four weeks that followed the initiation of adalimumab than between any of the subsequent visits. At Week 12, more than 15% of patients achieved clinical remission as defined by DAS28 < 2.6, and nearly double (28.9%) achieved a low-disease state (DAS28 < 3.2). Further, clinically important ameliorations in physical functions were observed in the first few weeks that followed the initiation of adalimumab therapy. These changes from baseline were maintained over time. Even patients who failed to satisfy ACR20 response criteria experienced significant improvements in most of the ACR core component measures with the exception of patient's global and pain evaluations.

The effectiveness of adalimumab against active RA that is unresponsive or only partially responsive to standard antirheumatic therapy demonstrated in this real-world trial confirms the results of other placebo-controlled studies in which adalimumab was administered either as monotherapy [13], in combination with MTX [11,12,14,15], or concomitantly with standard antirheumatic therapy [10]. Further, these results support earlier findings that adalimumab is efficacious even for patients who have failed or become intolerant to other TNF-α-antagonist therapies [24]. The data presented in the present analysis suggest that patients who are naïve to BDMARD therapy tend to achieve better disease status over a short period of time than patients with prior exposure to BDMARDs. While these results strongly support the use of adalimumab as a first line biological agent, adalimumab remains a viable option as a second line biological agent.

Adalimumab was well-tolerated in the CanACT study population with 87.8% completing the 12-week study. Only 39 of the 879 (4.4%) enrolled patients discontinued because of an adverse event. Most of the adverse events reported in this study were nonserious and considered to be either mild or moderate in intensity. Injection site reaction, which is frequently associated with subcutaneously administered agents, was the most commonly reported adverse event.

Nine patients in the study reported serious infections, resulting in an estimated rate of 2.4 events/100 patient-years. Similar rates have been observed during studies in which patients were treated with adalimumab plus MTX [11,14,15], or with standard antirheumatic therapy [10]. Patients treated with TNF-α antagonists are considered to be at increased risk of developing serious infections. However, the rate of serious infections reported in the present study was less than both the rate in the adalimumab clinical trial safety database (4.7/100 patient-years) and the rate in the general RA population as reported in the literature (3.1-9.6/100 patient-years) [25], a finding that may reflect the short duration of the study. There were no reported cases of tuberculosis during the study.

Congestive heart failure (CHF) was reported as a study-drug-related serious adverse event in 1 patient in the present study. This is in keeping with results from other clinical trials involving adalimumab [25,26], in which CHF has been described in patients both with and without a history of the disease. Proportionally, more patients with a history than without a history of CHF have reported this as an adverse event (7% vs. 0.3%). However, it should be noted that patients with a history of CHF were usually excluded from clinical trials. No cases of lupus or lupus-like syndrome occurred in patients in the present study, which is in accord with observations from previous clinical trials that demonstrated that these conditions are rarely associated with adalimumab [25,26].

The aforementioned results demonstrate that the safety profile of adalimumab is similar to that previously reported in controlled trials. In studies in which adalimumab was administered concomitantly with MTX [10-12,14,15], this combination was found to be safe and well-tolerated.

## Conclusions

In conclusion, the results of the present study demonstrate that adalimumab 40 mg given sc eow was effective in producing clinically important and statistically significant reductions in the signs and symptoms of disease. In addition, adalimumab therapy was associated with significant improvements in the physical functioning of patients with RA. The safety profile of adalimumab observed in this patient group was comparable with that of previous studies with this agent. Taken together, the current study supports the use of adalimumab 40 mg sc eow in the management of patients with RA who have not responded satisfactorily or who have proved intolerant to antirheumatic DMARD therapies.

## Competing interests

B.H. has received consulting fees or other remuneration from Abbott, Amgen, Bristol-Myers Squibb, Merck, Pfizer, Roche, and UCB. A.C. has performed clinical trial work for Abbott, UCB, Pfizer, Roche, Amgen, and Bristol-Myers Squibb. J.S. declares no competing interests. E.K. has received research grants or consulting fees/other remuneration from, or served on speakers bureaus on behalf of, Abbott, Amgen, AstraZeneca, Biotest, Bristol-Myers Squibb, Centocor, F Hoffmann-LaRoche, Genentech, Genzyme, Merck, Novartis, Nycomed, Pfizer, and UCB. B.G. is a full-time employee of Abbott and may hold stock or stock options.

## Authors' contributions

BH, AC, JS, and EK. were study investigators for the CanACT trial, and participated in its design and coordination. BG. participated in data interpretation. All authors read and approved the final manuscript.

## Pre-publication history

The pre-publication history for this paper can be accessed here:

http://www.biomedcentral.com/1471-2474/12/261/prepub
